# DNA methylation in necrotizing enterocolitis

**DOI:** 10.1017/erm.2024.16

**Published:** 2024-04-01

**Authors:** Lauren C. Frazer, Yukihiro Yamaguchi, Dhirendra K. Singh, Natalia S. Akopyants, Misty Good

**Affiliations:** Division of Neonatal-Perinatal Medicine, Department of Pediatrics, University of North Carolina at Chapel Hill, Chapel Hill, NC, USA

**Keywords:** DNA methylation, epigenetics, intestinal inflammation, necrotizing enterocolitis, neonatal, prematurity

## Abstract

Epigenetic modifications, such as DNA methylation, are enzymatically regulated processes that directly impact gene expression patterns. In early life, they are central to developmental programming and have also been implicated in regulating inflammatory responses. Research into the role of epigenetics in neonatal health is limited, but there is a growing body of literature related to the role of DNA methylation patterns and diseases of prematurity, such as the intestinal disease necrotizing enterocolitis (NEC). NEC is a severe intestinal inflammatory disease, but the key factors that precede disease development remain to be determined. This knowledge gap has led to a failure to design effective targeted therapies and identify specific biomarkers of disease. Recent literature has identified altered DNA methylation patterns in the stool and intestinal tissue of neonates with NEC. These findings provide the foundation for a new avenue in NEC research. In this review, we will provide a general overview of DNA methylation and then specifically discuss the recent literature related to methylation patterns in neonates with NEC. We will also discuss how DNA methylation is used as a biomarker for other disease states and how, with further research, methylation patterns may serve as potential biomarkers for NEC.

## Introduction

Gene expression is impacted by the nucleic acid sequence as well as non-genetically encoded modifications known as epigenetics. Epigenetic changes link extrinsic factors such as nutrition (Refs 1, 2, 3), infection (Refs 4, 5) and physiologic stress (Ref. 6) to gene expression patterns. These modifications are heritable and can thus serve as a transgenerational link between parental exposures and the genetic makeup of their children. Epigenetic changes can occur within the germ line or within individual cell types or organ systems and can regulate physiologic processes, such as development, as well as disease pathology. In addition, epigenetic modifications can downregulate the expression of damaged DNA (Ref. 7). Epigenetics has been well-studied in diseases that primarily impact adults, such as malignancies and inflammatory bowel disease (IBD); however, there is a paucity of research into their role in neonatal diseases.

In this review, we will discuss DNA methylation, which has been linked to early development and neonatal disease (Refs 8, 9, 10, 11, 12, 13, 14, 15, 16). We will specifically focus on the intestinal disease of prematurity, necrotizing enterocolitis (NEC). NEC is thought to result from immune cell hyperactivation and intestinal microbial dysbiosis (Refs 17, 18), which leads to intestinal epithelial damage and irreversible intestinal necrosis. Unfortunately, critical knowledge gaps in disease pathophysiology remain, and this has impeded progress in the identification of novel biomarkers and the implementation of effective therapies. Changes in DNA methylation patterns have only begun to be explored in the context of NEC. In this review, we will discuss recent studies identifying an association between methylation patterns in the stool and intestine with NEC (Refs 12, 13, 14, 15). Further research in this field may help elucidate important features of the pathophysiology of NEC, which is critical for improving diagnostic and therapeutic options for this devastating disease.

## Mechanisms of DNA methylation

**Figure 1.**
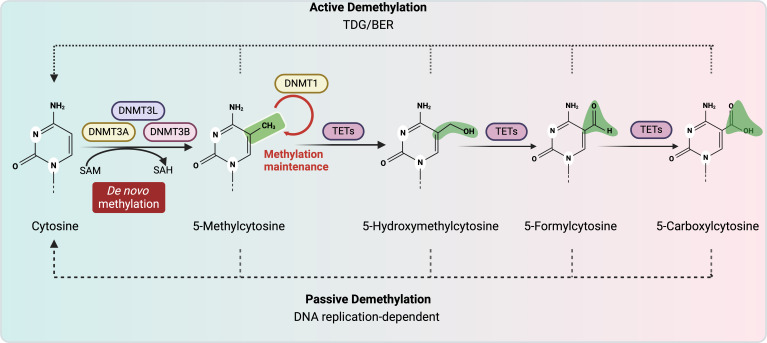
Human DNA methylation/demethylation pathways. DNA methylation is categorized as *de novo* and maintenance methylation. *De novo* methylation is characterized by the addition of methyl groups to previously unmethylated cytosines by the enzymes DNMT3A and DNMT3B, whose enzymatic activity is augmented by DNMT3L. Maintenance methylation is performed by DNMT1. SAM serves as the methyl donor for these reactions, resulting in the formation of SAH. Active demethylation is catalysed in a series of steps by TET enzymes followed by TDG and BER for the removal of the methyl group. Passive demethylation occurs when replication proceeds in the absence of DNMT function, thus leading to a reduction in the relative frequency of methylated DNA. DNMT, DNA methyltransferase; DNMT3L, DNA methyltransferase 3 like protein; SAM, S-adenosylmethionine; SAH, S-adenosylhomocysteine; TET, ten–eleven translocation; TDG, thymine DNA glycosylase; BER, base excision repair. Created with BioRender.com.

The three primary mechanisms of epigenetic regulation include (1) the attachment of non-coding RNAs such as microRNAs, (2) post-translational modifications of histone proteins and (3) DNA methylation. DNA methylation occurs when a methyl group is added directly to a cytosine nucleotide within a cytosine-guanine (CpG) dinucleotide sequence, generally on the fifth carbon of the cytosine (Fig. 1). DNA sequences that are enriched for CpG motifs, known as CpG islands, are hypomethylated and promote gene expression by regulating chromatin structure and transcription factor binding (Refs 19, 20). Analysis of the CpG content of human promotors demonstrated that 72% of promotors are located in CpG islands (Ref. 21), which points to a central role for differential DNA methylation patterns in the regulation of gene expression.

DNA methylation patterns are regulated by a balance of methylation and demethylation, and dysfunction in either of these pathways can result in dysregulation of gene expression and repair. The pathways involved in methylation and demethylation are detailed in Figure 1. The function of enzymes involved in DNA methylation falls into two general categories, maintenance methylation and *de novo* methylation (Ref. 22). Maintenance methylation is performed by DNA methyltransferase 1 (DNMT1), which copies DNA methylation patterns to daughter strands during DNA replication (Ref. 23). In addition, DNMT1 functions within DNA damage/mismatch repair pathways to protect cells from mutagenic events (Ref. 24). DNMT1 expression is essential during development as *Dnmt1* knock-out mice die *in utero* at mid-gestation (Ref. 23). In humans, mutations in DNMT1 are associated with the neurodegenerative disease hereditary sensory and autonomic neuropathy type 1 with dementia and hearing loss (Ref. 25). Patients with this condition have symptoms that progressively worsen after childhood, and brain atrophy is detectable on autopsy. These mutations are associated with an abnormal methylation pattern characterized by global hypomethylation and local hypermethylation (Ref. 25).

*De novo* methylation is performed by DNA methyltransferases 3A and 3B (DNMT3A and DNMT3B), and these enzymes are important in establishing DNA methylation patterns early in development (Refs 26, 27). Both DNMT3A and DNMT3B are necessary for survival as *Dnmt3a* knock-out mice die during weaning, and *Dnmt3b* knock-out mice as well as *Dnmt3a*/*3b* double knock-out mice die as embryos (Refs 26, 28). DNA methyltransferase 3-like (DNMT3L) protein enhances *de novo* methylation by DNMT3A and DNMT3B but does not possess its own enzymatic activity (Refs 29, 30). In humans, mutations in *DNMT3A* can lead to Tatton–Brown–Rahman syndrome, which is an overgrowth syndrome associated with intellectual disabilities (Ref. 31). Conversely, mutations in *DNMT3A* resulting in gain-of-function can lead to Heyn–Sproul–Jackson syndrome, a form of microcephalic dwarfism (Ref. 32). *DNMT3A* mutations have also been implicated in malignancies, including acute myeloid leukaemia and myelodysplastic syndrome (Ref. 33). DNMT3B is essential in the stabilization of pericentromeric satellite repeats, and mutations in this gene are associated with immunodeficiency, centromere instability and facial anomalies syndrome (Refs 26, 34). Clinically, DNA methyltransferase inhibitors are predominantly used to treat malignancies since reversible methylation changes are found in many cancers (Refs 35, 36). Mechanisms of action of these therapies includes reversing the inhibition of tumour suppressor genes caused by aberrant methylation and by inhibiting cellular replication (Ref. 36). Unfortunately, these agents can also have significant cytotoxicity and side effects (Ref. 36).

## Mechanisms of DNA demethylation

DNA demethylation is characterized as either active or passive. 5-Methylcytosine (5-mC) is the substrate for the 10–11 translocation (TET) enzymes, which are a family of methylcytosine dioxygenases that facilitate oxidation of 5-mC (Fig. 1) (Refs 37, 38). The modified forms of 5-mC, including 5-formylcytosine and 5-carboxylcytosine, serve as a substrate for thymine DNA glycosylase followed by base excision repair to yield unmethylated cytosine (Ref. 38). Alternatively, passive demethylation is not enzymatically mediated and occurs when DNA replication proceeds in the absence of DNMTs, thus leading to a dilution in the frequency of 5-mC. In humans, genetic syndromes resulting from mutations in demethylation enzymes are rare; however, TET3 deficiency has been associated with a syndrome characterized by developmental delay, abnormal growth, distinct facies and neurobehavioral difficulties (Ref. 39). In addition, reduced expression of TET3 in oocytes with pregestational hyperglycaemia or in human diabetes has been proposed as a mechanism that causes methylation-associated alterations in glucose tolerance in offspring (Ref. 40).

## The role of DNA methylation in early life

### Changes in methylation patterns during early development

DNA methylation patterns continue to evolve after birth. A longitudinal analysis of methylation patterns in saliva samples from infants at 6–52 weeks of age detected developmentally related changes in the methylation of 42 genes during this time frame (Ref. 8). These CpG methylation patterns have been found to be directly impacted by the gestational age (Ref. 9). A large meta-analysis involving samples from 3648 newborns in 17 different patient cohorts found that methylation of 8899 CpG motifs in 4966 genes in cord blood samples had a significant association with the gestational age of the neonate (Ref. 9). There was also an association between the methylation patterns in foetal cord blood, brain, and lung tissue at similar gestational ages (Ref. 9). Altered DNA methylation patterns have been associated with an increased morbidity risk in neonates born at 30 weeks gestation (Ref. 10). Specifically, methylation of 10 genes (8 with increased methylation, 2 with decreased methylation) had a statistically significant association with a neonatal morbidity risk score that included whether an infant had bronchopulmonary dysplasia (BPD), brain injury, serious infection or severe retinopathy of prematurity (Ref. 10). In addition, an epigenome-wide association study found significant differences in the DNA methylation patterns in cord blood of preterm neonates who subsequently developed BPD compared with those who did not develop lung disease (Ref. 11).

### DNA methylation in inflammation

Inflammatory stimuli, including bacterial (Refs 41, 42) and viral infections (Ref. 43), contribute to the epigenetic status of the genome. For example, in mice, infection of dams with the bacteria *Campylobacter rectus* was associated with foetal growth restriction and hypermethylation in the promoter of the insulin-like growth factor 2 (*Igf2*) gene in the placenta (Ref. 44). Furthermore, in vitro data and in vivo studies in neonatal mice found a complex interplay between the microbiome and antenatal steroid exposure on DNA methylation patterns (Ref. 45). Exposure of a foetal intestinal epithelial cell line to different bacterial species resulted in microbe-specific alternations in methylation patterns in vitro, and antenatal steroid administration resulted in changes in DNA methylation in foetal mice as well as significant differences in the intestinal microbiome composition at 2 weeks after birth (Ref. 45). In addition, analysis of intestinal epithelial cell DNA methylation patterns in mice found that the intestinal microbiome modulates the epigenomic and expression of genes linked to intestinal homeostasis (Ref. 46).

DNA methylation is also important in the regulation of innate immunity. For example, hypomethylation of the gene for the innate immune receptor Toll-like receptor 2 (TLR2) is associated with an increased pro-inflammatory response to bacterial peptidoglycan in bronchial epithelial cells from patients with cystic fibrosis (Ref. 47). Another TLR impacted by DNA methylation is TLR4, the innate immune receptor for lipopolysaccharide (LPS) expressed by Gram-negative bacteria. The responsiveness of TLR4 to LPS is regulated by DNA methylation and histone modification (especially acetylation) in intestinal epithelial cells when studied in vitro (Ref. 48). Murine studies examining *Tlr4* gene methylation in the intestine of germ-free (GF) and conventionally housed mice revealed a role for the microbiome in regulating *Tlr4* methylation status and gene expression (Ref. 49). They found that *Tlr4* methylation was reduced in the epithelial cells from the large but not the small intestine of GF mice, and there was an inverse relationship between methylation status and *Tlr4* expression (Ref. 49). Activation of TLR4 has been associated with the development of NEC and will be discussed in relation to NEC pathogenesis below (Refs 50, 51). Additionally, prenatal exposure to inflammation induced by *in utero* injection of heat-killed *E. coli* altered small intestinal DNA methylation patterns in the promoter regions of several genes involved in the TLR4 signalling pathway in a murine model (Ref. 52).

## DNA methylation in intestinal disease

Aberrant DNA methylation has been implicated in intestinal diseases in adults and children. For the purposes of this review, we will focus specifically on IBD, Hirschsprung's disease (HSCR), and NEC.

### Inflammatory bowel disease

Altered DNA methylation patterns have been implicated in the pathogenesis of IBD, which includes Crohn's disease and ulcerative colitis (Ref. 53). For example, mechanistic studies in mice supported the role of DNMT3A in regulating intestinal inflammation with an increased severity of experimental colitis and impaired epithelial cell regeneration in adult mice lacking DNMT3A specifically in their intestinal epithelium (*Dnmt3a^ΔIEC^*) (Ref. 54). These mice exhibit increased intestinal epithelial barrier permeability and decreased goblet cell numbers in the colon, and in vitro, intestinal enteroids from *Dnmt3a^ΔIEC^* mice have impaired wound healing and barrier formation (Ref. 54). From a clinical perspective, intestinal biopsies from patients with IBD were found to have significantly downregulated *DNMT3A* expression, which was also detected in intestinal organoids from these patients (Ref. 54). A meta-analysis of genome-wide association studies found that *DNMT3A* was an important risk locus for Crohn's disease (Ref. 55). Mice lacking DNMT1 in their smooth muscle cells have significantly impaired development of smooth muscle in their intestines (Ref. 56). These mice exhibit reduced weight gain, decreased intestinal length and impaired motility that leads to intestinal dilation and early mortality at approximately postnatal day 21 (Ref. 56). DNA methylation patterns in the peripheral blood of paediatric patients with Crohn's disease are influenced by disease status, with methylation patterns directly associated with levels of systemic inflammation reflected by increased expression of the inflammatory marker C-reactive protein (Ref. 57). More research into the clinical implications of disrupted DNA methylation in the setting of intestinal inflammation is needed to inform the development of new biomarkers and therapies.

### Hirschsprung's disease

HSCR is a congenital disease resulting from failed migration, proliferation or differentiation of rectal or colonic neural crest cells. This leads to the inability to pass stool, which can be life-threatening if megacolon or enterocolitis develops. The role of gene methylation patterns in HSCR has been explored in a limited number of studies (Ref. 58). A study of colonic tissue from patients with HSCR found that hypermethylation of the gene glial cell-derived neurotrophic factor alpha 4 (GFRA4) was significantly reduced in the colonic tissues of patients with HSCR (Ref. 59). GFRA4 is a member of a family of receptors expressed important in neuron survival and differentiation, which activate signalling via RET tyrosine kinase (Refs 60, 61). RET mutations are a common cause of HSCR (Refs 62, 63). In a separate study of human colonic tissues, increased expression of the G-protein coupled receptor, endothelin receptor type B (EDNRB), in the setting of DNA hypomethylation, was linked to HSCR development (Ref. 64). EDNRB is central to the pathogenesis of HSCR, as mice lacking *Ednrb* in their neural crest cells spontaneously develop a HSCR phenotype (Ref. 65). A broader whole genome methylation analysis found that enteric precursor cells obtained from patients with HSCR exhibited an overall pattern of DNA hypomethylation compared with controls (Ref. 66). These findings are in agreement with data demonstrating reduced expression of the DNA methyltransferase DNMT3B in neural progenitor cells in the setting of HSCR (Ref. 67) and increased expression of the demethylation enzyme TET1 in intestinal samples from patients with HSCR (Ref. 68). These findings support the role for a complex interplay between methylation and demethylation in regulating the formation of the enteric nervous system.

## DNA methylation in NEC

### Overview of the pathogenesis of necrotizing enterocolitis

**Figure 2.**
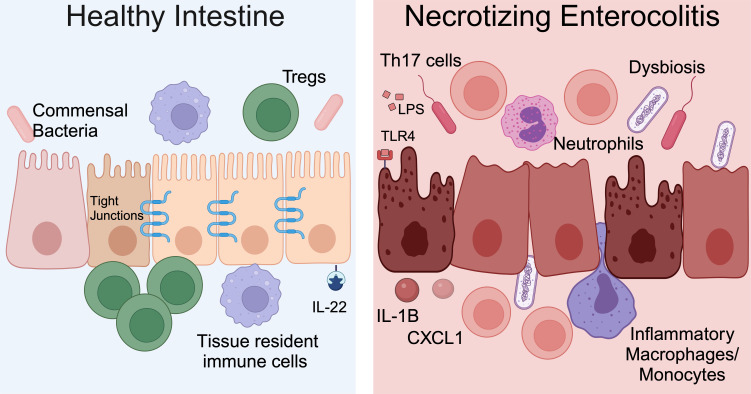
NEC pathogenesis. In the healthy intestine, the intestinal epithelium forms a selective barrier characterized by tight junctions between epithelial cells. There is an abundance of commensal bacteria. Tissue resident immune cells surveil the intestinal environment and regulatory T cells (Tregs) restrain inflammation. The cytokine interleukin (IL)-22 helps maintain homeostasis. During NEC, the microbiome is dysbiotic, LPS on Gram-negative bacteria activates TLR4 and the intestinal immune response is hyperinflammatory. Immune cells and inflammatory cytokines cause intestinal epithelial injury and impaired barrier function. Methylation patterns in the intestine and stool are altered for neonates with NEC. Created with Biorender.com.

NEC is a severe and potentially fatal intestinal disease that predominantly impacts preterm neonates. The incidence of NEC is as high as 8% for premature infants born weighing <1500 grams (Refs 69, 70, 71). Approximately 30% of infants with NEC require surgical resection of irreversibly damaged intestine, which is associated with a mortality rate of 30–50% (Refs 69, 70, 71). The aetiology of NEC is multifactorial and characteristic features include microbial dysbiosis, intestinal inflammation, ischemia and necrosis (Fig. 2) (Refs 17, 72, 73). Disruption of the intestinal epithelial barrier can lead to bowel perforation, sepsis and death. There are currently no targeted treatment options for NEC. Current therapies include discontinuation of enteral nutrition, broad-spectrum antibiotics and resection of necrotic bowel. Survivors of NEC are at high risk for long-term sequelae, including short-gut syndrome, poor growth and neurodevelopmental impairment (Refs 74, 75, 76). Early diagnosis of NEC is critical to improving outcomes, and implementation of clinically relevant biomarkers of disease, such as DNA methylation patterns, would allow for an immediate improvement in clinical care and facilitate clinical trials where accurate diagnosis and early implementation of novel therapies would be crucial.

### NEC and DNA hypermethylation

Ongoing research is focused on determining the disease mechanisms involved in the pathogenesis of NEC. Recent data point to a role for epigenetic modifications, such as DNA methylation, in NEC pathogenesis (Refs 12, 13, 14, 15, 16). Studies using whole genome bisulfite sequencing on epithelial cells isolated by laser capture microdissection from the colon or ileum of patients with NEC or at the time of reanastamosis (controls) found significantly increased methylation in samples from patients with NEC relative to controls (Ref. 16). This was most pronounced in the colon, where 38 809 CpG sites differed between the epithelium from surgical NEC samples and controls, whereas in the ileum, 652 CpG sites were hypermethylated. RNA sequencing on intestinal tissue from these patients revealed 1760 mRNAs with increased expression and 2596 mRNAs with decreased expression in the colon of patients with NEC relative to controls. For ileal tissue, there were 649 mRNAs with increased expression and 208 mRNAs with decreased expression during NEC. The authors determined that there were 7087 sites that were differentially methylated associated with genes with significantly different transcription between colonic samples from patients with surgical NEC and controls. Interestingly, 92% of differentially methylated sequences in promotors of genes that were downregulated in NEC were hypermethylated compared with 66% with increased expression (Ref. 16). The authors identified the transcription factor hepatocyte nuclear factor 4 alpha as the most statistically significant predicted regulator of the genes with differential promotor methylation in colonic samples. Variants in this gene have been previously associated with IBD (Refs 77, 78, 79). Examination of methylation patterns found that the promotor for G protein-coupled receptor 35 had the most significant degree of hypermethylation when comparing the samples from the colon of patients with and without NEC. Polymorphisms in this receptor are associated with IBD risk, and have been associated with protection in animal models of colitis (Ref. 80).

**Figure 3.**
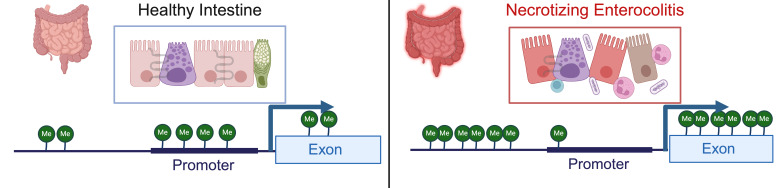
Methylation patterns in healthy intestine and NEC. NEC is characterized by intestinal inflammation, epithelial monolayer disruption, loss of specialized epithelial cells, microbial dysbiosis and bacterial translocation. DNA isolated from intestinal tissue from neonates with NEC has a distinct methylation pattern characterized by global hypermethylation except for CpG islands in promotor regions, which are hypomethylated. Created with BioRender.com.

Examination of methylation patterns using targeted genome-wide bisulfite sequencing performed on DNA from colonic tissue from patients with NEC and controls found that samples from patients requiring surgery for NEC were broadly hypermethylated (58.2%) compared with control samples (41.8%); however, CpG islands near promotors were hypomethylated in colonic tissue from neonates with NEC (43.6%) relative to controls (56.4%) (Ref. 13) (Fig. 3). This analysis also demonstrated a strong correlation between the degree of methylation of colonic tissues and enterocytes isolated from the colon from patients with and without NEC isolated by laser capture microdissection (Ref. 13). Comparison between RNA expression levels generated using RNA-sequencing and methylation patterns revealed that there were 2250 genes and 20 466 methylation sites that differed both in methylation and gene transcription between the colon of patients with NEC and controls (Ref. 13). There was a 56.35% correlation between expected gene expression and methylation status for NEC versus control (Ref. 13). Significant differences in methylation signatures in genes associated with regulating methylation, such as *DNMT3A*, *DNMT3B*, *TET1* and *TET3* were detected between tissue from neonates with and without NEC (Ref. 13). The highest rates of concordance were in promotor regions with (67.8%) and without (67.2%) CpG islands. They also found a direct correlation between methylation patterns in the colon and stool, with hypermethylation observed in the stool of patients with NEC relative to controls.

An additional study found that methylation of C-terminal domain small phosphatase like 2 (*CTDSPL2*) was significantly higher in the stool of neonates immediately prior to NEC onset compared with healthy preterm controls (methylation percent: NEC: 50.97% versus control: 17.02%, *P* = 0.047) (Ref. 12). This gene is involved in cell cycle regulation and inflammation (Refs 81, 82), but the phenotypic impact of increased methylation was not explored in this study (Ref. 12). In addition, these methylation changes were outside of a promotor region, and there was a high degree of overlap in the level of methylation between cases and controls (Ref. 12).

Signalling via TLR4 leads to increased intestinal injury during experimental NEC in mice (Refs 50, 51). Stool samples from infants obtained prior to NEC exhibited significantly higher methylation of the gene for TLR4 at CpG position 2, with a median of 75.4% methylation from the samples for infants with NEC and 69% for controls (Ref. 14). Similarly, colonic epithelial cells from neonates with NEC were found to have a 13% higher rate of methylation in the TLR4 promoter than in epithelial cells from control tissue (Ref. 16). Although additional studies are needed, these findings indicate that differential DNA methylation of TLR4 and TLR4-associated genes may be an important mechanism of intestinal inflammation in neonates.

## Methylation patterns as biomarkers

The identification of novel biomarkers for NEC for disease prediction or diagnosis is critically important for improving neonatal care. Currently, clinicians rely on abdominal imaging, non-specific symptoms and routine laboratory tests to diagnose infants with NEC. The inability to quickly and accurately diagnose infants with NEC can have devastating consequences, given the rapidly progressive nature of this disease. Unfortunately, there are no biomarkers for NEC that are used in clinical practice. DNA methylation patterns represent a promising target for new biomarker development for NEC, given the high level of DNA stability and literature showing that DNA methylation patterns are specific to their cell and tissue of origin (Refs 83, 84, 85). Here, we will discuss DNA-based diagnostic testing that has been implemented in other clinical arenas to demonstrate the feasibility of this approach and to inform further research related to biomarkers for NEC.

### Blood

Cell-free nucleic acid-based testing (cfDNA) has been implemented in prenatal care in the form of non-invasive prenatal testing (NIPT) since 2011 (Ref. 86). This test incorporates the detection of foetal DNA fragments in maternal plasma with various forms of sequencing to determine the risk of abnormal numbers of foetal chromosomes, such as in trisomy 13, 18 and 21 (Refs 87, 88, 89). This screening test permits the detection of foetal chromosomal anomalies as early as 9 weeks of pregnancy and was recommended in 2020 by the American Congress of Obstetricians and Gynecologists (ACOG) to be offered to all pregnant patients (Ref. 90). The clinical significance of this testing strategy is the non-invasive nature of obtaining samples from maternal plasma versus other more invasive methods such as amniocentesis and chorionic villus sampling, as well as the high sensitivity and specificity (Refs 89, 91). The use of circulating cfDNA was an advance on previous maternal blood tests that were faced with the technical limitations of reliably isolating DNA from the extremely small fraction of neonatal cells found in maternal blood, particularly early in pregnancy (Ref. 92).

Later studies expanded upon cfDNA by analysing cell-free RNA (cfRNA) in maternal plasma. Examination of cfRNA gene transcription in the plasma of pregnant women across all three trimesters demonstrated that it is possible to detect gene transcripts from foetal organs such as the liver and brain in maternal plasma (Ref. 93). These data indicate that further technological advances may lead to the utilization of foetal gene transcription patterns in maternal blood to predict and diagnose congenital anomalies. The potential clinical implications of this research were further supported by a pilot study examining cfRNA gene transcription patterns in three cohorts of pregnant women as a predictor of preterm birth (Ref. 94). The investigators determined that using a panel of seven cfRNA transcripts, they could identify women at an increased risk of preterm delivery with a mean area under the curve for the discovery cohort of 0.86 and 0.81 for the validation cohort (Ref. 94). These data indicate that cfRNA may be a clinically relevant tool to predict preterm delivery and is worth investigating as an adjunct to current prediction methods.

Biomarker studies into DNA methylation patterns in the peripheral blood found that overall peripheral blood white blood cell (WBC) methylation patterns are associated with a variety of malignancies, including colorectal cancer (CRC) (Refs 95, 96). In addition, gene-specific and global DNA methylation have been studied as a biomarker for cancer risk (Refs 97, 98, 99, 100, 101, 102). For example, methylation of the Septin 9 (*SEPT9*) promotor has been investigated in detail as a marker of CRC, and a meta-analysis found it to have good specificity (92%) but moderate sensitivity (69%) (Ref. 103). An FDA-approved test for CRC screening, named Epi proColon^®^, is a test for methylation of *SEPT9* DNA in plasma samples.

The use of WBC methylation patterns in the prediction of NEC is likely limited by difficulties in obtaining blood samples from critically ill neonates and their limited blood volumes; however, newer sequencing techniques requiring limited quantities of blood, such as from a heel-stick, would be feasible. In addition, WBC populations are characterized by different methylation patterns (Ref. 85), so it would be important to consider combining methylation analysis with the enumeration of leukocyte subpopulations via clinically utilized WBC counts or flow cytometry.

### Urine

Urine can be obtained using non-invasive methods and is a readily available source of DNA for methylation studies. Urine methylation patterns have been studied in the detection of CRC (Ref. 104). Regarding NEC, urine protein and microRNA expression levels but not methylation patterns have been studied as potential biomarkers (Refs 105, 106, 107). Thus, studies into the urine methylation patterns of preterm neonates for NEC and other diseases are warranted.

### Stool

Stool is utilized in biomarker studies in intestinal diseases because of the relative ease and non-invasive nature of sample acquisition; however, examining methylation patterns in stool can be technically challenging because of the abundance of bacterial DNA and the cellular degradation that occurs during passage through the intestinal tract. Despite these challenges, studies have identified altered methylation patterns in intestinal diseases such as CRC (Refs 108, 109, 110). In addition, stool DNA methylation testing in patients with IBD has been found to detect CRC and advanced precancerous lesions (Refs 111, 112). The strength of DNA methylation patterns in stool as a cancer biomarker is evidenced by the FDA approval of Cologuard^®^ as a screening tool for CRC, which incorporates abnormal gene methylation patterns into its non-invasive test (Ref. 113). This test has a 92.3% sensitivity and 89.8% specificity for CRC (Ref. 114) and is a widely utilized screening tool for individuals at average risk for CRC.

For NEC, the promise of DNA methylation patterns as a biomarker comes with the observation that stool methylation patterns correlate with those observed in intestinal tissue (Ref. 13). These findings indicate that methylation patterns in non-invasively acquired samples, such as stool, can reflect the neonatal intestinal inflammatory milieu. It is not feasible to obtain intestinal biopsies from critically ill neonates for diagnostic purposes, thus further research into methylation patterns in non-invasively acquired samples, such as blood, urine and stool, is essential to improving the clinical care of neonates with NEC.

## Conclusions

DNA methylation studies in NEC research are in their early stages (Refs 13, 14, 15, 16, 115, 116), but scientists and clinicians desperately need improved predictive and diagnostic tools for this deadly disease. The clinical implementation and widespread incorporation of cfDNA testing in prenatal care indicate that nucleic acid-based testing can have immense clinical utility. Although the NIPT does not incorporate methylation analysis, this testing provides proof of principle for the utility of cell-free techniques. In regard to NEC, stool DNA methylation signatures correlate with colonic methylation patterns for patients with NEC, indicating that stool methylation status may be a clinically useful tool to investigate the inflammatory milieu of the neonatal intestine (Ref. 13). This technology has the potential to improve our understanding of disease pathophysiology and contribute to the development of novel biomarkers and therapeutics. Targeting epigenetic machinery would represent a new treatment strategy for NEC, and developing novel, improved therapies for this disease is critical for this vulnerable patient population.
